# Extension of mepolizumab injection intervals as potential of saving costs in well controlled patients with severe eosinophilic asthma^☆^^☆^

**DOI:** 10.1016/j.waojou.2022.100703

**Published:** 2022-10-01

**Authors:** Georg Bölke, Xunliang Tong, Torsten Zuberbier, Jean Bousquet, Karl-Christian Bergmann

**Affiliations:** a Institute of Allergology, Charité – Universitätsmedizin Berlin, Corporate Member of Freie Universität Berlin and Humboldt-Universität zu Berlin, Berlin, Germany; b Department of Respiratory and Critical Care Medicine, Beijing Hospital, National Center of Gerontology, Beijing, PR China; c Fraunhofer Institute for Translational Medicine and Pharmacology ITMP, Allergology and Immunology, Berlin, Germany

**Keywords:** Biologics, Severe asthma, Mepolizumab, Injection intervals

## Abstract

**Background:**

Present guidelines recommend a life-long therapy with mepolizumab in patients suffering from severe eosinophilic asthma as several studies proved the disadvantages of treatment cessation. This study evaluated the possibility of extending the dosage intervals of mepolizumab in those patients with severe eosinophilic asthma after being well controlled.

**Methods:**

Eighteen patients diagnosed with severe eosinophilic asthma were started on treatment with mepolizumab in regular 4-week intervals. Symptom control was measured using the asthma control test (ACT) and pulmonary function test every 3 months. The amount of oral corticosteroids needed to maintain symptom control was monitored at every visit. After achieving good symptom control, defined as well controlled ACT ≥20, injection intervals were prolonged from 4 up to 6 to 8 weeks. The evaluation of this data was approved by the ethics committee.

**Results:**

ACT and pulmonary function values significantly improved after initiating therapy with mepolizumab on a regular 4-weekly injection interval. After extending the dosage intervals, both ACT and pulmonary function remained on a stable level without significant changes during the follow-up visits for 1 year. Median dosage of prednisolone declined significantly in the studied group under mepolizumab therapy and stayed on a low level during the follow-up visits with only a single patient using prednisolone after 1 year.

**Conclusion:**

In patients with fully or well controlled eosinophilic asthma treated with mepolizumab extending the dosage intervals between the injections up to 8 weeks bears the potential to save costs for the health care system.

## Introduction

Around 3.6% of the asthmatic population suffer from severe asthma.^1^ Severe asthma is classified as a subtype of uncontrolled asthma despite high dose long-acting beta-2-agonists (LABA)/inhaled corticosteroids (ICS) and treatment of contributory factors.^2^ Current guidelines recommend considering biologic therapies as a next additional therapy step if the patient meets the requested criteria. Around two-thirds of these patients are eligible to be treated with mepolizumab, a humanized monoclonal antibody binding circulating interleukin-5 (IL-5) and therefore inhibiting the development and recruitment of eosinophils.^3^^,^^4^ Mepolizumab has proven its beneficial effects and safety in several real-life studies improving pulmonary function and disease control and reducing exacerbation rates.^5^, ^6^, ^7^, ^8^

After reaching a stable state, further strategy remains unclear. With improved well-being and reduced symptom severity the first step would be a reduced dosage or a cessation of oral corticosteroid (OCS) therapy. After stopping oral treatment, decreasing from high dose to moderate dose ICS would be the next preferred option. A further reduction or a complete cessation of inhaler therapy is not supported by current guidelines. Thus, the biologic itself remains as an adjustment option. Discontinuation of mepolizumab worsens asthma control and increases the risk of acute asthma exacerbations.^9^^,^^10^ Another possibility may be a dose reduction. However, with missing substantial data for clinical outcomes after dose reduction of mepolizumab on one hand, and the impracticability of a reduced dose administration in the increasing use of patient self-administration with prefilled autoinjectors on the other hand, this does not seem a promising approach.

The remaining option would be an extension of the injection intervals. For therapy with omalizumab, an anti-immunoglobulin E monoclonal antibody, interval extensions were shown to be a reasonable approach to maintain asthma control.^11^ During the COVID-19 pandemic a position paper of several allergic medical associations considered a prolonged injection interval of biologics to reduce patient to physician contact.^12^

This study investigates the effects of interval extension of mepolizumab on asthma symptoms in patients achieving good disease control after initiation of anti-IL-5 therapy.

## Methods

### Patients and study design

This retrospective study used data from the outpatient clinic of the [blinded in this version] collected between 2016 and 2020. Selected patients needed to have uncontrolled severe eosinophilic asthma despite high dose ICS/LABA to initiate treatment with mepolizumab. Patients received mepolizumab according to official product information with a fixed dose of 100 mg subcutaneously every 4 weeks.^13^

Initial injections (visit −1) were given at the outpatient clinic, later injections were either continued at the clinic or self-administered via autoinjector or pre-filled syringes by the patient itself as approved by the European Medicines Agency (EMA).^14^ Besides elimination of exacerbations and withdrawal of OCS, one of the treatment goals was reaching a stable symptom status defined by the asthma control test (ACT) with ≥20 points.^15^^,^^16^ The ACT consists of 5 questions evaluating disease control over the past 4 weeks. Each question has a score from 1 to 5 points with higher scores relating to better asthma control. An overall score from 20 to 25 points represents a well-controlled asthma. Some patients achieved a controlled status (ACT at least 20 points, no exacerbations since start of therapy with mepolizumab) and felt so well that they prolonged injection intervals in contrast to current guidelines. This date was defined as visit 0. Patients were informed about this off-label use and the potential risk of worsening asthma and gave written informed consent. Intervals were regularly extended to a 6-weekly course, but intervals up to 8 weeks were accepted according to patient requests and continuous symptom stability.

Disease control was evaluated every 3 months (visit 1 after 3 months, visit 2 after 6 months, visit 3 after 9 months, visit 4 after 12 months) for 1 year using the asthma control test. Pulmonary function was monitored by spirometry measuring the forced expiratory volume in 1 s (FEV1) and the forced vital capacity (FVC). Daily intake of oral corticosteroids for asthma therapy was registered at each visit.

Patients missing the last follow-up visit 1 year after the start of interval extension conducted a postponed visit (visit 5). Every other patient was able to participate at visit 5, though it was not obligatory.

The evaluation and publication of data reported in this manuscript was approved by the institution's ethics committee.

### Statistical analysis

Statistical analyses were computed with IBM SPSS Statistics 27 for Windows and Microsoft Excel for Microsoft 365. A p-value < 0.05 was accepted as statistically significant. Data are described as median with range if not stated otherwise. Because of the small sample size, only non-parametrical tests were used. Differences between characteristics at date of first injection and start of interval extension were analyzed with the Wilcoxon signed-rank test. During the follow-up visits, differences were calculated with the Friedman test.

## Results

### Demographics

From a total of 64 patients treated with mepolizumab in the outpatient clinic, 18 patients undergoing interval extension were identified in this retrospective analysis. Demographics are shown in Table 1. Most common comorbidities were nasal polyposis (55.6%), arterial hypertension (50%) and aspirin-exacerbated respiratory disease (33.3%).

**Table 1:** Demographic data at the date of start with Mepolizumab therapy. All data are shown as median with range in brackets. IgE – immunoglobulin E

Sex	6 (33.3%) female
Age at first injection (years)	57 (30–77)
Body mass index (kg/m^2^)	25.4 (20.0–37.2)
IgE (kU/L)	397.5 (28–1294)
Eosinophils (/μL)	375 (33–1230)

Length of time between beginning of mepolizumab treatment and start of interval extensions was 20.3 (13–81) weeks. Planned 3 monthly follow-up visits were conducted after 13.3 (12–17) weeks at first visit, 26.9 (24.7–31.1) weeks at second visit, 40 (34.6–41.9) weeks at third visit, 52.6 (43.3–68.9) weeks at fourth visit and 84 (80.3–109.6) weeks at fifth visit.

Visits 1–4 were attended by 16, 16, 15, and 16 patients, respectively, for each visit and 5 patients at visit 5.

At beginning, intervals were prolonged to a 6-weekly course for everybody but 1 patient, who started with an 8-weekly interval upon specific patient request. At the fourth visit, 4 patients (22.2%) had been changed to an 8-weekly interval while the others remained on the 6-weekly course. Intervals were never shortened when prolonged once.

### Asthma control test und pulmonary function tests

**Fig. 1:**
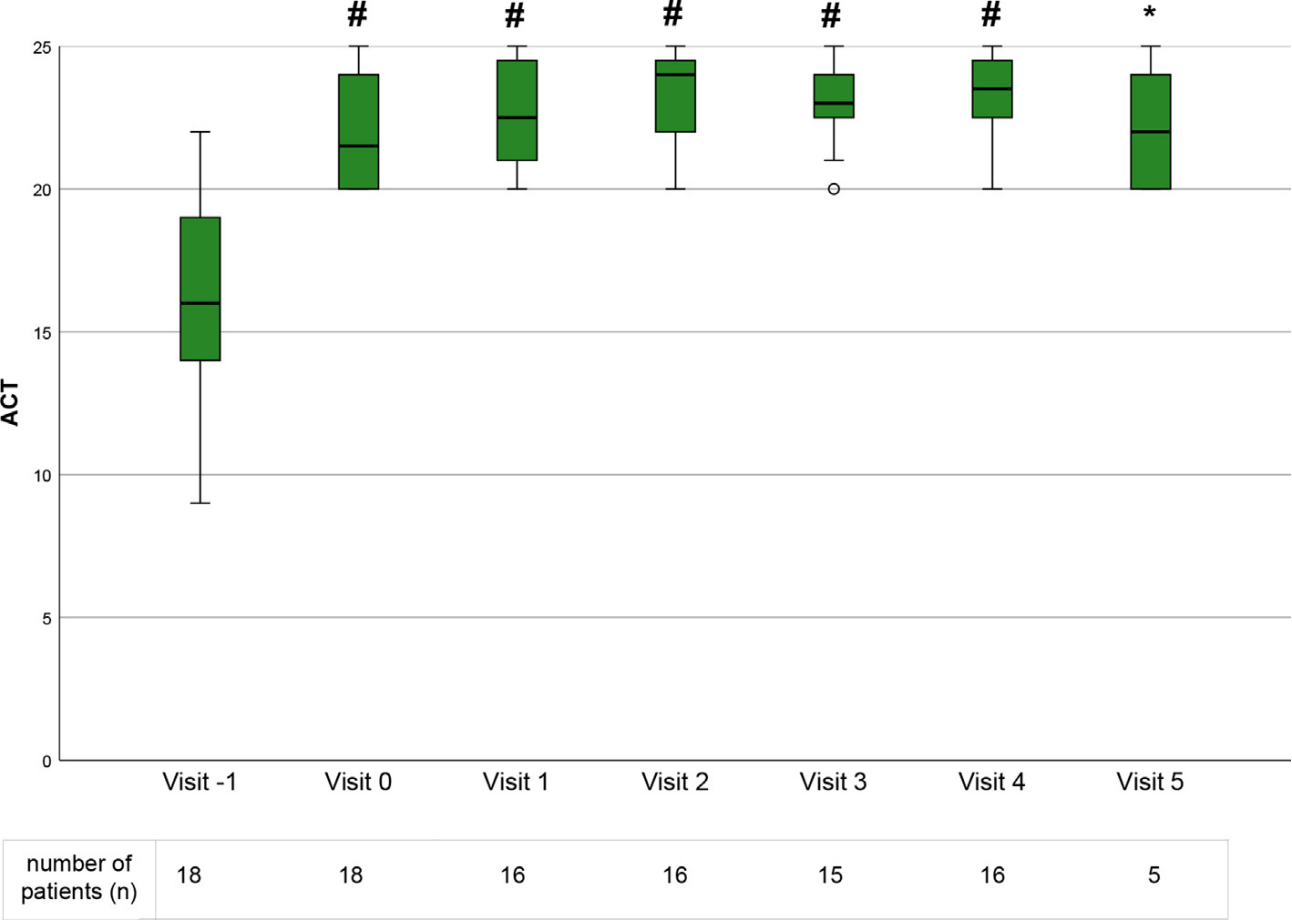
Asthma control test results at each visit. Data is displayed as boxplots with medians. Outliers are presented as degree sign (o). Significant differences in comparison to visit −1 are marked as asterisk (∗) for p < 0.05 and hash (#) for p < 0.001. ACT, asthma control test

**Fig. 2:**
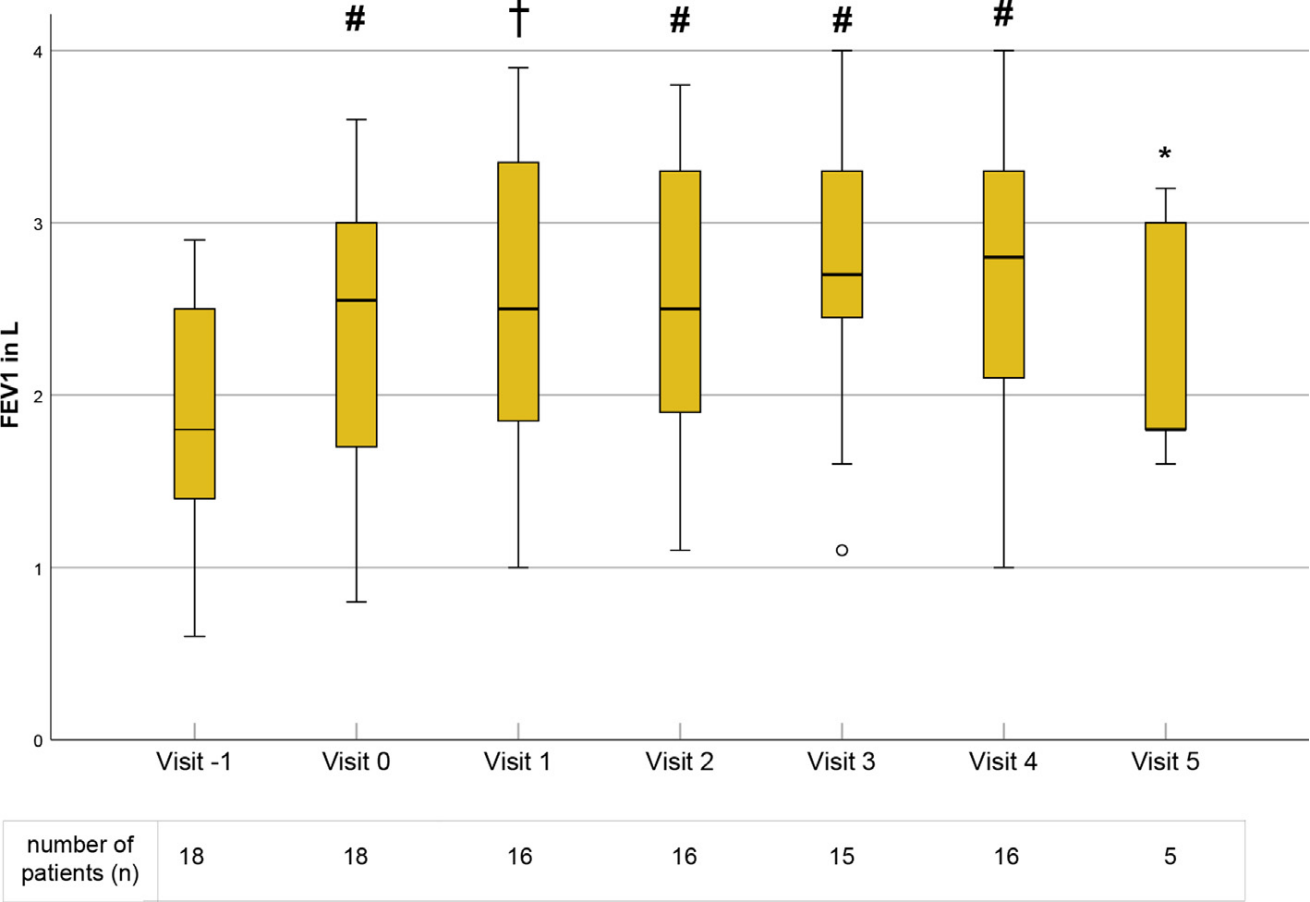
FEV1 results at each visit. Data is displayed as boxplots with medians. Outliers are presented as degree sign (o). Significant differences in comparison to visit −1 are marked as asterisk (∗) for p < 0.05, dagger (†) for p < 0.01, hash (#) for p < 0.001. FEV1 – forced expiratory volume in 1 s in Liter

Asthma control test results significantly improved from 16 (range 9–22) to 21.5 (20–25) points after initiating mepolizumab therapy (p < 0.001). Equally, FEV1 increased from 1.8 L (0.6–2.9) to 2.6 (0.8–3.6) (p < 0.001), FEV1% from 59 (33–78) to 74.5 (44–91) (p < 0.001) and FVC from 3.5 (1.2–5.0) to 3.8 (1.4–5.6) (p = 0.01) at visit 0. Both ACT and pulmonary function values stayed on a stable level without significant changes during the follow up visits (Table 2, Fig. 1, Fig. 2).

**Table 2:** Pulmonary function and asthma symptom test values during the study period. Data is shown as median with range in brackets. Significant differences in comparison to visit −1 are marked as asterisk (∗) for p < 0.05, dagger (†) for p < 0.01, hash (#) for p < 0.001. ACT, asthma control test; FEV1, forced expiratory volume in 1 s (L – in liter, % - predicted in percent), FVC, forced vital capacity (in liter)

	Visit −1	Visit 0 (interval extension)	Visit 1	Visit 2	Visit 3	Visit 4	Visit 5
ACT	16 (9–22)	21.5 (20–25)^#^	22.5 (20–25)^#^	24 (20–25)^#^	23 (20–25)^#^	23.5 (20–25)^#^	22 (20–25)∗
FEV1 (L)	1.8 (0.6–2.9)	2.6 (0.8–3.6)^#^	2.5 (1.0–3.9)†	2.5 (1.1–3.8)^#^	2.7 (1.1–4.0)^#^	2.8 (1.0–4.0)^#^	1.8 (1.6–3.2)∗
FEV1%	59 (33–78)	74.5 (44–91)^#^	70.5 (57–90)†	73 (47–94)^#^	76 (58–94)^#^	80 (57–94)^#^	81 (47–87)∗
FVC (L)	3.5 (1.2–5.0)	3.8 (1.4–5.6)∗	3.9 (1.5–5.6)†	3.9 (1.5–5.5)^#^	4.0 (1.6–5.5)†	3.9 (1.5–5.4)^#^	3.9 (2.1–4.8)

### Use of oral corticosteroids

**Fig. 3:**
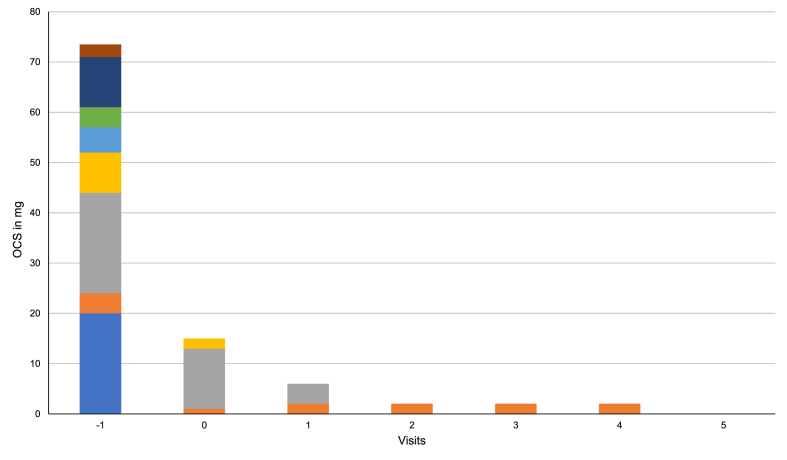
Daily dose of OCS at every visit. Each color represents an individual patient. OCS, oral corticosteroid

Eight patients (44%) needed OCS (prednisolone) at start of therapy with mepolizumab. At the beginning of the interval extensions, 3 of 8 patients were still on OCS. Median dosage in this patient group declined from 6.5 mg (range 2.5–20 mg) to 0 mg (range 0–12 mg) (p = 0.012). Two of these patients had ceased OCS at the first and second visit, respectively (Fig. 3). During follow-up visits, median OCS dosage remained at 0 mg. Only 1 patient continued OCS therapy with a low dosage of 2 mg at visit 4 (no visit 5 data available for this patient). Importantly, the low dose OCS was not taken for asthma control but to suppress rheumatic joint pain. No patient was initiated on therapy with OCS during the period of this study.

## Discussion

While several studies proved the disadvantages of discontinuation of mepolizumab therapy in patients suffering from severe eosinophilic asthma to the authors knowledge no data exists evaluating the effects of a reduced cumulative dosage by either reducing the single dosage or extending the intervals between the injections of mepolizumab. In this retrospective study, the injection interval of mepolizumab was extended from 4 to 6–8 weeks in 18 patients. Both asthma control and lung function remained stable over the 12-month observation period. None of the patients suffered from an asthma exacerbation in the observed period.

A pharmacokinetic study reported the terminal half-life of mepolizumab after subcutaneous injections as 22 days.^17^ However, even 12 weeks after the last administration of mepolizumab, plasma concentration levels could still be measured, though being most likely not clinically relevant. Also, blood eosinophils measured throughout the study did not reach the baseline level again. Another pharmacokinetic and dynamic study conducted with healthy male subjects found a maximum decrease in blood eosinophil count 29 days after injection of 75 mg mepolizumab intravenously (equivalent to 100 mg subcutaneous).^18^ Interestingly, blood eosinophils remained on comparable low levels until day 57, approximately 8 weeks, before increasing again but not returning to baseline until the last day of assessment (day 85). The authors suggest that a single dose of mepolizumab can suppress blood eosinophil count for at least 2 months, supporting the results of our study that kept patients on stable asthma symptom control without significant differences between the 6- or 8-weekly intervals. Similar results were obtained in a study comparing the pharmacokinetic of the newly available liquid formula used in autoinjectors against the initially developed lyophilized formulation.^19^ Both formulas shared similar pharmacokinetic profiles without significant differences, providing authenticity for our study where both options of therapy were used. On day 57 and 85 (approximately 8 and 12 weeks) after drug injection, blood eosinophils still were declined 50–60% compared to baseline and plasma concentrations of mepolizumab were measurable.

Though no study exists observing the effects of an interval extension of mepolizumab in asthma patients, some data sustaining our findings can be derived from studies evaluating the cessation of the biologic therapy. The COMET trial evaluated the effects of ceasing therapy with mepolizumab in individuals treated with 100 mg subcutaneously for at least 3 years.^20^ Patients were randomized to receive either continued mepolizumab injections or placebo and monitored for 52 weeks every 4 weeks. Subjects without further mepolizumab therapy experienced significantly more often clinically relevant exacerbations, a shorter time of period until declined asthma control (measured with the Asthma Control Questionnaire-5 [ACQ-5]) and increased blood eosinophil counts over the 52-week observation period. While significant differences in blood eosinophil counts were already found 8 weeks after the last mepolizumab dosage, exacerbation rates and impaired asthma control showed significant differences only 16 weeks after the last injection. In other words, disease control seemed to maintain for even 12 weeks after stopping mepolizumab therapy.

Though our study may not be directly comparable as median time until start of prolonged intervals was only 20 weeks and the ACQ-5 as the tool of symptom control specifically asks for symptoms in 1 week recall as the ACT recalls the past 4 weeks, we also did not observe loss of asthma control up to 8 weeks after the last injection. Further interval extensions might be not beneficial. Two studies evaluating the effects of stopping mepolizumab treatment reported increased exacerbation rates, rising blood and sputum eosinophils and less controlled asthma symptoms according to ACQ-5 scores 12 weeks after the last injection.^10^^,^^21^

At least 2 trials exist treating patients with mepolizumab injections in prolonged intervals. Roufosse et al evaluated the efficacy of mepolizumab in hypereosinophilic syndrome.^22^ After establishing a stable symptom state dosage interval could be extended up to ≥24 weeks (median 12.8 weeks). However, the used dosage of 750 mg mepolizumab intravenously makes the results difficult to compare to ours.

Another small case study reported an interval extension from 4 to 8 weeks for 2 asthmatic patients with chronic eosinophilic pneumonia.^23^ Both young patients had elevated blood eosinophils (730/μL and 1130/μL). Oral corticosteroids could be quit after 7 and 10 months of mepolizumab treatment. Interval was extended 12 and 14 months after beginning of treatment and stable state without disease progression or exacerbation have been observed for 1 and 2 years, respectively. Again, a direct comparison to our results cannot be drawn as the treated disease features different characteristics, but it supports our observations that patients with an eosinophilic disease with stable disease control under therapy with mepolizumab might not need injections on a 4-weekly base to maintain symptom control.

As this study was analyzed using retrospective data, it has important limitations. First, the number of included patients is small (n = 18). Secondly, due to its real-life character no matched control group maintaining the regular 4-weekly injection interval could be included in this study. Moreover, during the follow-up visits none of the patients experienced an asthma exacerbation. Thus, no statement can be made about the rate of exacerbations under extended dosing intervals. Possibly a longer observation period with more patients included yields further information in this relevant knowledge gap in biologic treatment.

## Summary

Biologics for Severe Asthma are in use as “add-on therapy”. In many cases, they are extremely successful in avoidance of exacerbations, reduction of oral steroids, and increase of FEV1 and asthma control. In real life a part of patients who achieved all these points are reducing also their inhaled steroid and the use of long-acting betamimetics. Some of them use only their biologic as self-application despite the current guideline recommendations. Our data suggest that interval extension of mepolizumab injections from 4 to 6 up to 8 weeks might be an option in patients with well-controlled asthma seeking to reduce their therapy burden. This mode may be seen as patient-individual therapy.

## Abbreviations

ACQ5, Asthma Control Questionnaire-5; ACT, asthma control test; FEV1, forced expiratory volume in one second; FEV1%, forced expiratory volume in one second predicted in percent; FVC, forced vital capacity; ICS, inhaled corticosteroid; IL-5, interleukin-5; LABA, long-acting beta-2-agonist; OCS, oral corticosteroids.

## Acknowledgements

None.

## Funding

No funding to declare.

## Availability of data and materials

The data used and analyzed for this study is available from the corresponding author on reasonable request.

## Author contributions

KCB designed the study. XT and KCB acquired the data. GB performed the statistical analysis. GB, XT, TZ, JB and KCB evaluated and interpreted the data. All authors participated in drafting and revising the article and approved the final manuscript for submission.

## Ethics statement

The study was approved by the Ethics committee of the Charité – Universitätsmedizin Berlin (No. EA1/098/18). All research was conducted in accordance with the Declaration of Helsinki.

## Submission declaration

All authors reviewed the final manuscript and provided consent to its publication.

## Competing interest declarations

TZ reports consultancy from Bayer Health Care, FAES, Novartis and Henkel, grants/grants pending from Novartis and Henkel, payments for lectures from AstraZeneca, AbbVie, ALK, Almirall, Astellas, Bayer Health Care, Bencard, Berlin Chemie, FAES, HAL, Leti, Meda, Menarini, Merck, MSD, Novartis, Pfizer, Sanofi, Stallergenes, Takeda, Teva, UCB, Henkel, Kryolan and L’Oréal, organizational affiliations as committee member in WHO-Initiative “Allergic Rhinitis and Its Impact on Asthma” (ARIA), member of the Board in German Society for Allergy and Clinical Immunology (DGAKI), head of European Centre for Allergy Research Foundation (ECARF), president of Global Allergy and Asthma European Network (GA2LEN), member of Committee on Allergy Diagnosis and Molecular Allergology, World Allergy Organization (WAO), outside of the submitted work. JB reports personal fees from Chiesi, Cipla, Hikma, Menarini, Mundipharma, Mylan, Novartis, Purina, Sanofi-Aventis, Takeda, Teva, Uriach, other from Kyomed-Innov, outside the submitted work. KCB reports personal fees for lectures from ALK, AstraZeneca, Allergopharma, Almirall, Bencard, Chiesi, GSK, HAL, LETI, Lofarma, Mundipharma, Novartis, Sanofi, non-financial support as Chair of German Pollen Information Service Foundation, personal fees and non-financial support from consultant physician for ECARF, personal fees and non-financial support from Advisory Board member of AstraZeneca, ECARF, GSK, Robert-Koch- Institute Berlin (Vice chairman Public Health), Sanofi, outside the submitted work. GB und XT report no competing interests.
